# Socioecological systems analysis of potential factors for cholera outbreaks and assessment of health system’s readiness to detect and respond in Ilemela and Nkasi districts, Tanzania

**DOI:** 10.1186/s12913-023-10263-7

**Published:** 2023-11-15

**Authors:** Baraka L. Ngingo, Zaina S. Mchome, Veneranda M. Bwana, Augustino Chengula, Gaspary Mwanyika, Irene Mremi, Calvin Sindato, Leonard E.G. Mboera

**Affiliations:** 1 Department of Applied Sciences, Mbeya University of Science and Technology, Mbeya, Tanzania; 2 National Institute for Medical Research, Mwanza Research Centre, Mwanza, Tanzania; 3 National Institute for Medical Research, Amani Research Centre, Muheza, Tanzania; 4 Department of Microbiology, Parasitology and Biotechnology, Sokoine University of Agriculture, Morogoro, Tanzania; 5 National Institute for Medical Research, Dar es Salaam, Tanzania; 6 National Institute for Medical Research, Tabora Research Centre, Tabora, Tanzania; 7 SACIDS Foundation for One Health, Sokoine University of Agriculture, Morogoro, Tanzania

**Keywords:** Cholera, Potential factors, Readiness, Detection, Response, Tanzania

## Abstract

**Background:**

Cholera outbreaks are a recurrent issue in Tanzania, with Ilemela and Nkasi districts being particulary affected. The objective of this study was to conduct a socio-ecological system (SES) analysis of cholera outbreaks in these districts, identifying potential factors and assessing the preparedness for cholera prevention and control.

**Methods:**

A cross-sectional study was carried out in Ilemela and Nkasi districts of Mwanza and Rukwa regions, respectively in Tanzania between September and October 2021. A SES framework analysis was applied to identify potential factors associated with cholera outbreaks and assess the readiness of the districts to cholera prevention and control.

**Results:**

Ilemela is characterised by urban and peri-urban ecosystems while Nkasi is mainly rural. Cholera was reported to disproportionately affect people living along the shores of Lake Victoria in Ilemela and Lake Tanganyika in Nkasi, particularly fishermen and women involved infish trading. The main potential factors identified for cholera outbreaks included defecation in the shallow ends and along the edges of lakes, open defecation, bathing/swimming in contaminated waters and improper waste disposal. The preparedness of both districts for cholera prevention and response was found to be inadequate due to limited laboratory capacity, insufficient human resources, and budget constraints.

**Conclusion:**

People of Ilemela and Nkasi districts remain at significant risk of recurrent cholera outbreaks and the capacity of the districts to detect the disease is limited. Urgent preventive measures, such as conducting considerable community awareness campaigns on personal hygiene and environmental sanitation are needed to alleviate the disease burden and reduce future cholera outbreaks.

**Supplementary Information:**

The online version contains supplementary material available at 10.1186/s12913-023-10263-7.

## Background

Cholera is an acute diarrhoeal disease caused by eating or drinking food or water contaminated with *Vibrio cholera* O1 or by direct inter-human exposure, such as contact with hands contaminated with faeces from a cholera patient [1]. It is endemic in more than 50 countries in the world putting 1.3 billion people at risk [2]. Globally, it has been reported that approximately 2.8 million cases and 95,000 deaths occur annually [2]. Cholera has been largely eliminated from high-income countries through water and sewage treatment over a century ago, but remains a significant cause of illness and death in many African and Asian countries [3]. The number of cholera cases and the proportion of deaths remains higher in Africa than elsewhere in the world [3]. This is associated with weak health systems, inadequate sanitation and hygiene and insufficient access to safe drinking water that favours cholera transmission [4, 5]. Improving access to potable water, sanitation and hygiene (WASH) are critical to reducing Africa’s cholera burden [6].

Since 1974 when cholera outbreak was reported for the first time in Tanzania [7], the country experiences spatioal progression of cholera outbreaks almost every year with over 250,000 cases and 13,078 deaths [8, 9]. From 15 to 2015 through 17 March 2019, 33,437 cases including 552 deaths have been reported across all 26 regions of the United Republic of Tanzania translating to an overall country-level case fatality rate of 1.7% [10].High-risk areas experiencing cholera outbreaks in Tanzania include the coastal and central regions and those on ecosystem of Lakes Victoria, Tanganyika and Nyasa [8, 11–13]. Cholera has remained endemic in Tanzania with outbreaks associated with devastating consequences at individual, community and health syststem’s levels [9]. To effectively prevent and control the disease outbreaks, there is a need to improve understanding of the mechanisms and processes underlying pathogen emergence, spread and persistence.

Recent political, socioeconomic and cultural shifts resulting from attempts to address the social ecological crisis affects natural communities (animals, human and other organisms) and ultimately their pathogens need to be understood [14]. The socio-ecological systems analysis looks at disease incidence and outcomes as a bio-geo-physical unit and its associated social actors and institutions rather than purely statistical terms [15]. It attempts to address the questions regarding space, time and vulnerability of the population in question, issues that are critical in the design of appropriate interventions. Therefore, the socio-ecological model provides a framework for exploring the interaction of individuals, their health, and their physical and social environments at multiple levels of a health problem and their interdependency [16].

Despite the significant role of socio-ecological analysis in understanding disease emergencies [17, 18], only a few studies have applied socio-ecological systems (SES) framework analysis to infectious disease outbreaks in Sub-Saharan Africa [19, 20]. The objective of this study was to carry out a SES analysis of cholera outbreaks in the Ilemela and Nkasi districts of Tanzania to identify potential factors and assess preparedness and response of the districts in its prevention and control.

## Methods

### Study area

**Fig. 1:**
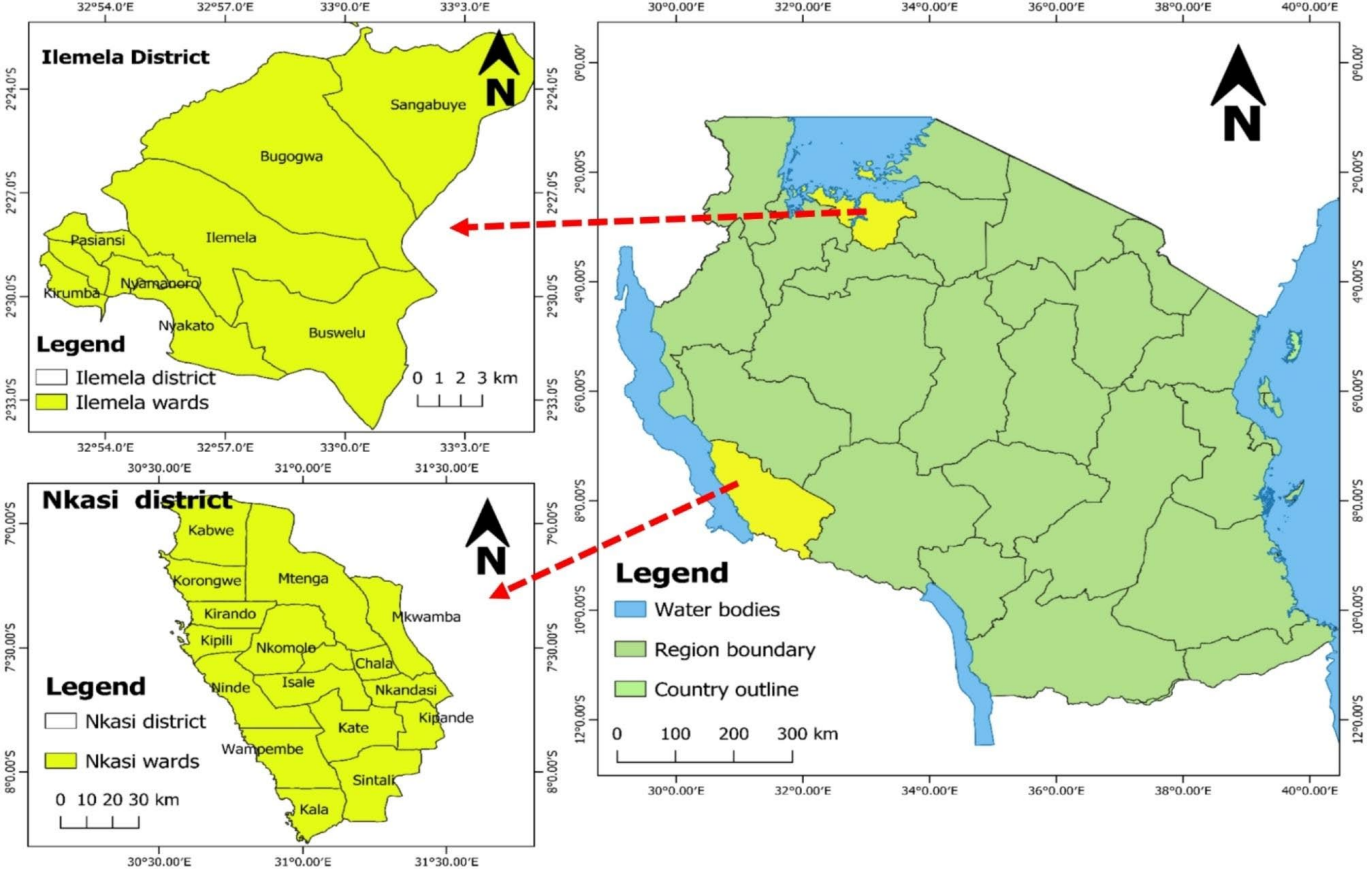
Map of Tanzania showing the study districts

This study was carried out in Ilemela and Nkasi districts in Tanzania between September and October 2021. Ilemela district is located between latitude 2.4485^0^ South and 32.9663^0^East in Mwanza region in north-western Tanzania (Fig. 1). The district is administratively divided into 19 wards with a human population of 480,779 and a population density of 1,347 per km^2^ with an annual growth rate of 2.6%. The district is characterised by both urban and peri-urban ecosystems. In Ilemela, the main economic activities are subsistence and large-scale crop agriculture, pastoralism, industry, fishing, and business. Ilemela is served by 58 health facilities, which include four (4) hospitals ( one (1) government-owned, one (1) parastatal-owned, one (1) faith-based and one (1) privately-owned); Five (5) health centres (four (4) government-owned and one (1) parastatal-owned); and 49 dispensaries (14 government-owned, and 35 privately-owned).

Nkasi district is in Rukwa region of western Tanzania lying between latitudes 7.800^0^South and longitude 30.7833^0^ East (Fig. 1). It has an area of 13,124 km^2^, of which 9,375 is land and 3,749 is covered by water. The district has a human population of 281,200 translating into a population density of 16.0 per km^2^. Administratively, the district is divided into 28 wards. Nkasi is largely rural, with over 90% of its population undertaking subsistence farming, livestock farming and fishing. The climate is warm throughout the year and rainfall ranges from 750 to 1200 mm per annum. The major crops include maize, millet, beans, and cassava.

### Study design

This was a cross-sectional study that applied a socio-ecological system (SES) framework analysis as a lens (through which to examine and reflect a topic of interes) to understand the multiple level factors associated with cholera outbreak in the study districts. It was carried out through desk reviews and consultative workshops with selected members of the district Council Health Management Team (CHMT). The aim was to explore and gain an understanding of the linkage and interplay between the human system and the natural system in a two-way feedback relationship affecting cholera outbreak dynamics. For the human system, we assessed the ecological factors associated with the occurrence of cholera and resilience at the district level. The themes (representing participants’ perceptions and opinions on cholera outbreak dynamics and capacity of the district to detect and respond) in the SES framework analysis tool (checklist) included ecosystem descriptor, human settlement type, economic activities, agricultural production system, and housing structure [20]. Others included infrastructure, actors influencing disease events, policies and external shocks to the health system. In this study, we considered the major components of the SES framework namely, social, economic, and political settings; resource systems; governance systems; resource units; actors; interactions; implementation outcome; and related ecosystems. Application of this theory-based framework to understand the multi-faceted and interactive effects of different actors and environmental factors on the outcome of interest involved five nested, hierarchical levels of the socioecological model, which are individual, interpersonal, community, organizational, and enabling environment.

### Data collection

The study participants were selected purposively from of the list of district health officials representing different responsibilities in disease prevention, control and surveillance programmes/activities. The selection of participants was also based on their position, experience and expertise on issues relevant to this research. They included District Health Secretary, District Health Officer, District Health Management Information System focal person, District Integrated Disease Surveillance and Response focal person, District Vector Control Officer, District Laboratory Technologist, Environmental Health Officer, and District Medical Officer.

The data collection process was implemented in the respective disticts through a participatory rapid appraisal method to explore practices related to prevention and control of cholera outbreaks. The workshop participants were oriented to the SES framework analysis, objectives of the study and expected outcomes. They were taken through data collection tools for a common understanding of different fields related to SES analysis [20]. Each participant was then provided with a printout of a SES analysis matrix, which included issues to be addressed regarding the internal and external shocks for the occurrence and risk management of cholera. The process involved group work and plenary discussions to identify potential factors and construct perceived thematic causal-relationship explanations of cholera outbreaks in the study districts [20]. We sought to understand the enablers and barriers affecting the health system’s efficiency the risk management of cholera outbreaks in the study districts. Additionally, detailed hand-written notes were made by the researchers to document key content of the discussion and any other observations arising during the workshops. One of the investigators carried out the extraction of data on cholera from the District Health Information System database covering a period of seven years (2015–2021). This timeframe was selected to reflect the most recent cholera outbreaks in the country including the most extensive the occurred in 2015. In addition, we were limited to this period based on completeness of the dataset besides the interest to explore, as part of readiness, if the health system would have detected signals of cholera outbreaks prior to the 2015 outbreak.

### Data analysis

Following the completion of each workshop, the researchers read the filled SES matrices, line by line to identify the potential codes and developed an initial coding scheme. Additional data from the hand-written field notes were used to clarify and expand the participants’ views. Two researchers manually coded all materials thematically utilizing the guidance by Braun and Clarke’s [21]. A series of deductive codes were created, based on the four topics on the research instrument (SES matrix). These included the clinical competence of healthcare providers, diagnostic capacity, surveillance system in place and its functionality, and control interventions available. The analysis implemented amd the findings of this study serves as an exploratory baseline data to guide mitigations against the disease prevention and control and future hypothesis-driven studies.

## Results

### Drivers of cholera outbreaks

Generally, in Ilemela, most of people in the fishing community were at risk of contracting cholera. Fishermen, who are mostly men, are at risk of contracting cholera than other segments of the population because of their practice of drink untreated water directly from the lake during their daily fishing activities. Women engaging in buying and selling raw fish from the lake shores was another vulnerable group reported. Additionally, being responsible for preparing food for the family, all women, in general, were considered to be at an increased risk of exposure to cholera pathogens on raw fish from the lake. Children were reported to be exposed to the disease from their practice of swimming in the shallow end of the lake.

The study revealed a number of potential factors fuelling cholera in Ilemela community, including: (i) Improper toilet waste disposal during rain/wet season particularly by people residing in the up-hills and squatter settlements; (ii) People’s habits to defaecate in the shallow end of the lake while swimming; (iii) The community members’ habits of drinking water from untreated sources including lake, wells, streams; (iv) Involvement in fishing, which expose people (particularly men) to pathogens from contaminated lake water; (v) People’s habits of bathing/swimming in the shallow end of the lake. Other factors included people’s practices of not using the toilets and the cultural norm among the local people that forbids toilet sharing with in-laws, which in the end influences the use of bush/ a field for open defecation. Furthermore, most of the community members in Ilemela were reported to have negative attitude towards boiled or treated water and therefore, do not boil water before drinking as they believe that it loses taste, and if treated using chlorine, it smells bad. Additionally, the community members consider boiling or treating water as an expensive exercise given that it involves buying charcoal/firewood and/or chlorine.

In Nkasi, cholera was reported to affect mainly people living along the shores of Lake Tanganyika. Fisherfolks living in fishing camps as they practise open defecation due to the absence of latrines in such temporary settlements were described to be at the risk of contracting cholera. Others people at risk of contracting cholera included people who are swimming in the lake, fish traders, particularly those who move to and from different countries in the region such as the Democratic Republic of Congo, Zambia, and Burundi; families with no toilets; children as they are unable to take care of their personal hygiene. People living or working along the shores of Lake Tanganyika including traders and those consuming traditional brews from local bars operating under poor hygiene conditions were also described to be at risk.

The potential factors reported to be associated with cholera in Nkasi included (i) socio-cultural behaviour associated with improper disposal of human faces; (ii) swimming in Lake Tanganyika; (iii) consumption of local brews; (iv) practices of not using toilets; (v) consumption of unwashed fruits; (vi) people’s habits of defecating in the shallow end of the Lake Tanganyika while swimming; (vii) the community members’ habits of using untreated water from the lake for domestic purposes; and (viii) people’s habits of not treating drinking water based on the belief that when treated the water loses taste and/ smells bad; use of untreated water for drinking and low coverage of improved latrines. Other factors included people’s habits of using a bush or a field for open defecation; lack of / and improper use of toilets among community members; and the presence of traditional breweries operating under poor hygienic conditions. There was a perception among the participants that the mango season, coupled with the rainy season incrases the risk of choleraoutbreaks. During mango seasons, most of cities and markets receive more mangoes than consumption capacity which end up rotting due to the perishable nature of mangoes. Also because of shortage of clean water many people eat unwashed mangoes and if the mangoes are contaminated with Vibrio cholerae, there is chance of spreading the pathogen.

**Fig. 2:**
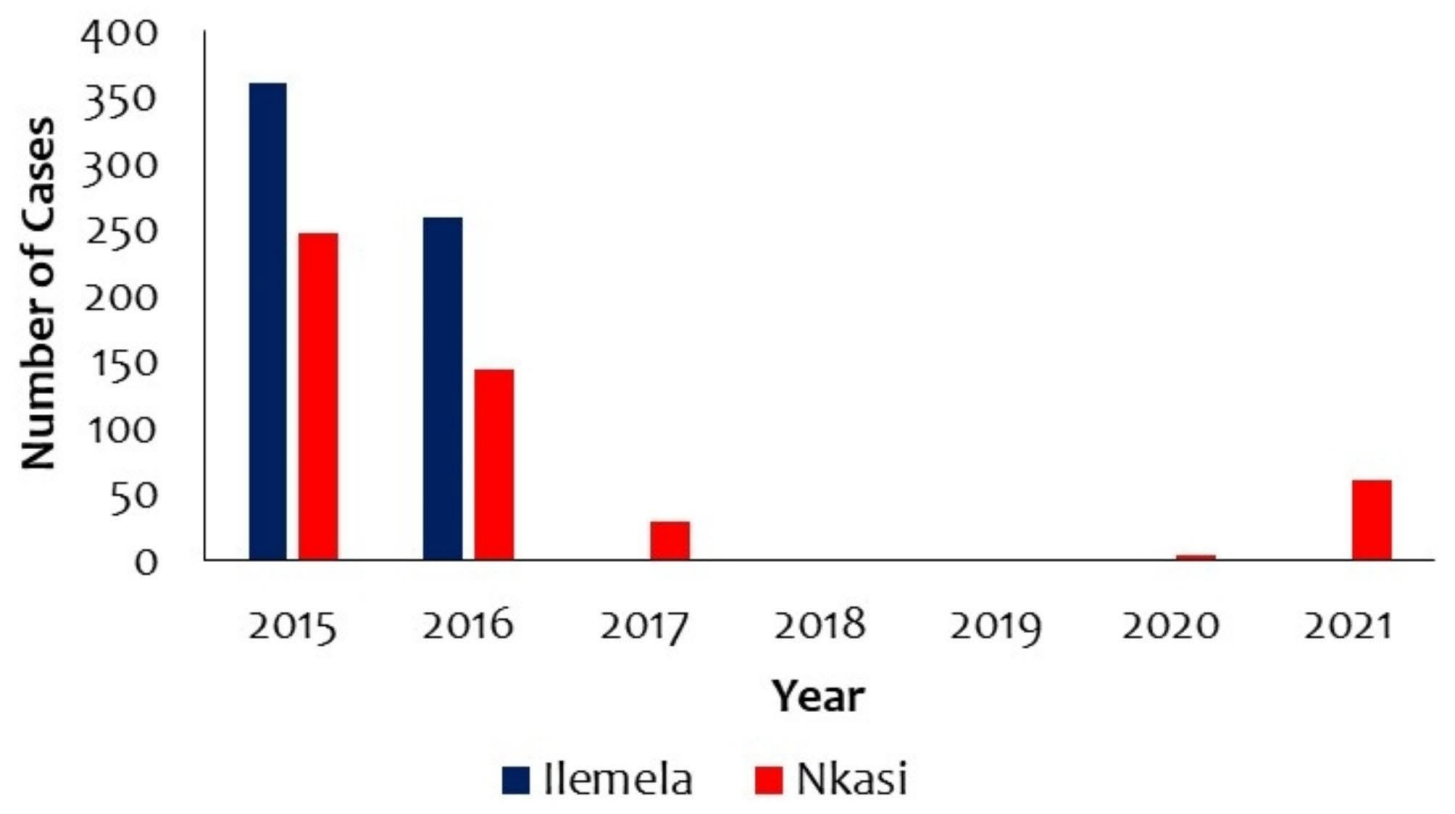
Cholera cases in Ilemela and Nkasi district, 2015–2021

Data extracted from District Health Information System 2 (DHIS2) showed that 42,004 cholera cases were reported between 2015 and 2021 (Fig. 2). Of these cases, 618 (1.5%) and 492 (1.2%) were from Ilemela and Nkasi, respectively. The two districts experienced large epidemics in 2015.

### Cholera preventive and control measures

In llemela, preventive measures against cholera included health education and promotion on construction and proper use of toilets/latrines as well as proper waste disposal. There was also an ongoing programme known as the “triggering programme” that aimed to sensitize the community to observe hygiene and appropriate waste disposal and water treatment. This initiative was being implemented by the Ministry of Health with the aim of making people feel and have knowledge that drinking untreated water from sources such as lakes, wells, and streams is risk for their health and to self-motivate to construct latrines/toilets and avoid open defecation. The programme involved the community and the community health workers in identifying possible risk of cholera incidences (e.g., families who have no toilets, and people who practice open defecation). In addition, the programme aimed to promote environmental and personal hygiene via various channels including community meetings and the provision of education related to cholera at the health facilities.

In terms of prevention and management of cholera, the district has facilities to test water safety, including the detection of coliforms; the presence of emergency rooms and diarrhoea and treatment rooms in each health facility. Other preventative measures include treatment /disinfection of public water sources for domestic use; and enforcement of relevant laws. There are Beach Management Units in Ilemela which are responsible for water cleaning and enforcing proper use of and maintenance of toilets among fisherfolks. These units are sponsored by Lake Victoria Environmental Management Project.

Preventive measures against cholera implemented in Nkasi included conducting massive campaigns to raise community awareness of the importance of personal hygiene and environmental sanitation including construction, proper use of toilets and handwashing, mass promotion of the use of “water guards” (a dilute sodium hypochlorite solution used as a point-of-use treatment for household drinking water) in treating water for drinking, improve water sanitation via increased access to improved latrines in the community and clean/safe water. Health promotion and education were being provided through local radios. The district has promoted the formation of a village health committee in each village to oversee environmental sanitation and hygiene. The village health committees also ensure that no activities at the lake that expose people to the risk of contracting cholera (such as swimming and bathing) are taking place. Additionally, village leaders regularly check the availability of latrines in each household and pose fines for those without the facilities. This is one of bylaws used in Tanzania as the means of forcing people have their latrines. It is a mandatory that each household must have a latrine andthere is a promotion of construction and proper use of improved toilets. The community health workers provide quarterly reports on the number of toilets per village. The government constructed toilets on the Lake Tanganyika islands and educated the community on their proper use. There was a countrywide campaign to promote the construction of improved toilets and their proper use .

### Cholera outbreak preparedness

Cholera preparedness in Ilemela was reported to be weak. Cholera standard case definition (SCD) and case management guidelines were available. A functional referral mechanism was in place. Though human resources were available, it was described to be inadequate. In fact, the district lacks health workers with capacities specifically for cholera outbreaks. When there was a cholera outbreak, health experts were alyways mobilized from healthcare facilities. Laboratory capacity in relation to cholera was inadequate.

Some of the challenges in cholera case detection and response/prevention and control reported are summarised in Table 1. Others included inadequate human resource, lack of poor infrastructure including roads. In Nkasi, although the participants claimed to have adequate SCD for cholera, case management guidelines were available in only a few health care facilities. There were no isolation centres for cholera cases. Thus, during a cholera outbreak, the district would establish temporary isolation centres at the health facilities. In case a village has no health facility, the government buildings are designated as alternative isolation centres. The inadequate human resource was identified as a major challenge in outbreak preparedness. In terms of diagnostic capacities, cholera diagnosis is centralized, with the sample collected being shipped to Sumbawanga Regional Hospital for testing and confirmation. On the other hand, Nkasi mentioned non-government organization (Plan International Project and Mfiril Lake Tanganyika), water and sanitation authority, community leaders, religious leaders, political leaders as their collaborators in cholera control and prevention activities.

**Table 1:** Capacities in cholera detection and response by district

Variable		Ilemela	Nkasi
Clinical competence	Standard case definition	Available	Inadequate
	Human resource	Inadequate	Inadequate
	Treatment facilities	Available	Available
	Diagnostic facilities	Not available	Not available
Diagnostic capacity	Guidelines	Available	Available
	Inadequate	Not available	Not available
Surveillance system	Surveillance system	Case-based	Case-based
	Outbreak response team	Not available	Available
	Data analytical capacity	Adequate	Available
	Data sharing	Done	Done
	Use of surveillance data	Planning	Planning
Control/Prevention strategies	Contingency plans	Available	Available
	Budget for health emergencies	Not available	Not available
	Diarrhoea treatment corner	Available	Not available
	Health education and promotion campaigns	Available	Available

### Surveillance systems

In Ilemela, the surveillance system for cholera was case-based (systematic reporting of newly diagnosed cases). The health system also supports timely incidence notification from each health facility. However, the surveillance system was challenged by a number of issues including (i) lack of transportation system for health promotion activities at community level, and for case management and surveillance team; (ii) shortage of human resources; (iii) shortage of related medical supplies/facilities i.e., cholera beds, protective gears; (iv) poor infrastructure including shortage of special/designated spots for managing cholera patients; and (v) lack of budget for outbreak response. Surveillance data management was reported to be fairly good, with routine data quality control being done (e.g., cross-checking the received data from various sources, and rectifying errors, when necessary). The district officials claimed to have adequate data analytical capacity. Surveillance data was shared quarterly with other stakeholders in the community (community health experts, environmental / animal health experts) through meetings. The health experts also regularly update the community on the top 10 diseases in the council. The data is used for planning, budgeting, and ordering medical equipment/supplies including those related to cholera.

Preparednes and Response Contingency plans for cholera outbreaks were available. The district usually set an annual budget for health emergencies. The budget is usually used for staff training and the purchase of related medical supplies. In each health facility, there was a diarrhoea and treatment corner (DTC), as well as an emergency room. However, in most cases, these rooms were small in size to cater for the needs during cholera outbreaks. Additionally, the rooms were been used for other activities in the absence of emergencies, thus taking a long time to prepare them when an outbreak occurs. The community members were always involved in implementing preventive measures/initiatives against cholera. From the participants, the partners or collaborators in cholera response in the district include private health facilities and non-governmental and community-based organizations, as well as community leaders and community health workers. For instance, Amref Health Africa, formerly AMREF (Africa Medical and Research Foundation) was involved in the promotion and provision of handwashing facilities, and facilities for emptying wastewater, particularly for the hard-to-reach communities in the uphill squatters with poor water disposal infrastructures. Lake Victoria Environmental Management Project supports the construction of toilets for fishing communities. The other important actors are the Mwanza Urban Water Supply and Sanitation Authority.

The surveillance response team for outbreaks in Nkasi was available and functional, whereby the surveillance committee members meet quarterly. However, the operation of the team is challenged by: (i) a lack of isolation centres (ii) unavailability of funds for timely response; and (iii) poor infrastructure (facility building, means of transport, and roads). Surveillance data were considered reliable and timely, however, sometimes the timely notification of events is challenged by poor network connection given that some of the facilities are located in remote / hard to reach villages. The district has a good data analytical capacity. The data analysis is done by the district data manager in collaboration with the Health Management Information System (HMIS) Coordinator. Data collected was reported to be shared with other stakeholders such as water and the environment sectors, and is used for budgeting health services, training, resource and staff allocation. The district had an outbreak contingency plan (updated in 2020), but the availability of a budget for outbreak response is a challenge.

### Key actors influencing cholera events

In the Ilemela district, various actors have been identified to impact cholera events either positively or negatively. Visitors coming for business, particularly fish buying, were found to potentially contribute to cholera outbreaks, while local and external stakeholders collaborated with the district health leadership to mitigate cholera through the efforts of ward health officers, community health workers, local government leaders, politicians, Mwanza Urban Water Supply and Sanitation Authority, and health agents like Beach Management Units and community-based organizations.

In the Nkasi district, key actors involved in cholera control include community health officers, local leaders, and religious leaders, who play a crucial role in implementing health-promoting initiatives. Politicians, particularly Councils, and elders are actively involved in providing health education during outbreaks. Additionally, non-governmental organizations like Plan International, United Nations Children Fund, and Mfiri Lake Tanganyika Project are also significant stakeholders contributing to cholera control efforts.

## Discussion

Despite the lesson learned from previous cholera outbreaks in Tanzania since 1974, cholera remains to be a persistent public health problem. This shows that effective prevention and control of cholera cannot be achieved from only thestudies conducted traditionally with a focus on pathogen-host interaction and statistical-based inferences, but would largely benefit from a holistic approach that considers an improved understanding of different-level factors, including health system capacity and readiness, as a bio-geo-physical unit. The basic idea of socioecological system analysis (SES) is to understand health problems in a system thinking approach and linking together ‘human system’ and ‘natural system’ in a two-way feedback relationship. It helps to understand and generate hypotheses regarding the interactions between humans, pathogens and the environment s. Moreover, the readiness of national and sub-national levels in cholera prevention and control is important in the designing and planning of appropriate intervention measures.

Ilemela district is characterised by both urban and peri-urban ecosystems. The main economic activities are subsistence and large-scale crop agriculture, pastoralism, industry, fishing, and business. Nkasi district is largely rural, with over 90% of its population undertaking subsistence farming, livestock rearing, and traditional fishing. Very interesting, one-third of the villages in Nkasi are located along the shores of Lake Tanganyika, and areas that was previously reported to be vulnerable to cholera outbreaks [22]. Therefore cholera being an outbreak prone disease in Ilemela and Nkasi may be explained by the presence of large bodies of water, which can act as reservoirs for cholera pathogens when contaminated [3]. The finding that people drink untreated water and use lake water for domestic use is alarming, swimming and bathing need to be avoided to contribute to cholera outbreak prevention. Studies have shown that Oral cholera vaccine is effective in cholera prevention in endemic communities [23]. It is recommended that Tanzania government use cholera oral vaccine for prevention of cholera in Ilemela, Nkasi and many other districts that reports cholera cases continuously.

In the SES framework analysis, common factors for cholera outbreaks in Ilemela and Nkasi included; involvement in fishing activities, habit of drinking untreated water from lakes (Lake Victoria for Ilemela and Lake Tanganyika for Nkasi), lack or improper use of latrines, the habit of bathing/swimming in the shallow end of lakes, open defecation and improper waste disposal. These findings are consistent with those found in previous research conducted in Tanzania, Malawi, Uganda and the Democratic Republic of Congo [11, 22, 24, 25]. All these factors can greatly hinder effective cholera prevention and control strategies if not taken into consideration.

On assessing the outbreak preparedness or readiness and capacity in responding to cholera outbreaks, both districts reported a weak readiness, limited diagnostic capacity and dependence on a regional level. Although cholera standard case definition and case management guidelines were available, human resources were inadequate and laboratory capacity to test and confirm cholera at the district level was limited hindering timely outbreak response. In both districts, there was an alternative of shipping samples to other labs at the regional level for testing and confirmation. Weak preparedness of the district health system is also greatly associated with a lack of budget allocation and or disbursement. This calls for concerted efforts to improve resource availability and training. The existing evidence shows that in many cholera-prone settings, the ability to use culture-based diagnostic approaches is restricted by insufficient or lack of appropriate laboratory capacity [26]. This is linked to the fact that in many African countries, there are problems in staff management, lack of trained laboratory staff, unavailability of laboratory supplies, challenges with the storage and transport of samples, unreliable reporting and lack of overall financial support [26].

In both districts, the surveillance system for Cholera is case-based. Generally, the performance of surveillance systems in the study districts was satisfactory. Data collected is shared between stakeholders and used for Planning, Budgeting and Ordering medical equipment and supplies. Although available Health systems in Ilemela district are found to supports timely incidence and notification, the effectiveness of the surveillance system is hampered by lack of enough budget, lack of transport systems, inadequate human resources and inadequate infrastructure. This also calls for concerted efforts to improve resource availability and training.

Most countries rely on hospital-based surveillance of diarrhoeal disease to compute and monitor the cholera burden. Countries in the WHO-Africa region adopted the Integrated Disease Surveillance and Response strategy in 1998 to enhance surveillance for priority public health diseases, conditions and events, including cholera [27]. In many places in rural Tanzania, accessibility to health facilities and affordability are likely to be challenges in seeking health care. On average, 45% of the population in Tanzania live within 5 km to the nearest health care facility. The accessibility of healthcare services in Tanzania has been associated with inadequate fund, skilled healthcare staff, poor communication, transport infrastructure and ability to pay for transport. Moreover, there is disparity in the distribution of the facilities [28]. Moreover, availability of medicines and qualified human resources were the major factors on the preference for accessing health care services [28, 29].

Regarding cholera control interventions, this study found successes of the health system in strategies to control cholera outbreaks. The health systems of the study districts have managed to engage the community, conduct health education (though conducted during outbreaks only), promotion on construction and proper use of latrines as well as proper waste disposal. In addition, districts have reported success in involving other stakeholders including non-governmental organizations such as Plan International, United Nations Children Fund and Mfiri Lake Tanganyika Project. If these strategies will be sustainably implemented, the burden of cholera outbreaks in respective districts will be lowered.

Based on the cross-sectional nature of the study implemented in two districts and our approaches in data collection, it worth to note that the study was not designed to explain the causal relationship, and any attempt to make the results transferable to other settings should be made with caution. The findings from this study provides opportunity to generate testable hypotheses for more informative and wider-area studies to improve understanding of multiple-level factors interactions and their influence on cholera outbreaks. There was limitede descriptive analysis of the data in this study. Hence, future studies using appropriate descriptive analysis methodology are recommended, to establish whether potential vulnerable groups are at risk of being infected by cholera, such as fishermen, who are mostly mem, and women engaging in buying and selling raw fish from lake shores.

## Conclusions

In conclusion, this study highlights the heightened risk of cholera outbreaks in the communities of Ilemela and Nkasi districts. The findings reveal weaknesses in the ability of the district health systems to detect and respond effectively to cholera outbreaks. Urgent measures are required to strengthen the health systems in the study area, enabling early detection and rapid response to such outbreaks. Additionally, there is a crucial need to implemeent community-based control and prevention measures through robust campaigns to combat the spread of cholera effectively.

### Electronic supplementary material

Below is the link to the electronic supplementary material.

Supplementary Material 1

## Data Availability

The datasets generated and analysed during the current study are not publicly accessible. Howerver, interested parties may obtain the datasets from the corresponding author upon a reasonable request.

## Abbreviations

CHMT: Council Health Management Team
CHWs: Community health workers
DTC: Diarrhoea and treatment corner
DHIS2: District Health Information System 2
HMIS: Health Management Information System
SCD: Standard case definition
SES: Socio-ecological systems
WASH: Water, sanitation and hygiene
WHO: World Health Organisation
