# Supplementary material for: Socioecological systems analysis of potential factors for cholera outbreaks and assessment of health system’s readiness to detect and respond in Ilemela and Nkasi districts, Tanzania
Source: BMC Health Serv Res. 2023 Nov 15;23:1261. doi: 10.1186/s12913-023-10263-7 (PMC10652585; doi:10.1186/s12913-023-10263-7)
Supplement: Supplementary file 1 — Supplementary Material 1 [file 12913_2023_10263_MOESM1_ESM.docx]

**SOCIO-ECOLOGICAL SYSTEM ANALYSIS TOOL (CHECKLIST) FOR DIFFERENT THEMES TO ASSESS POTENTIAL RISK FACTORS OF CHOLERA OUTBREAK AND READINESS OF THE HEALTH SYSTEM IN THE DETECTION AND RESPONSE**

| ***Study area profile*** | | |
| --- | --- | --- |
| Ecosystem descriptor |  | |
| Administrative district |  | |
| Human settlement type and name |  | |
| Peridomestic animal community farmers |  | |
| Main economy process |  | |
| Agriculture production system |  | |
| Affected community |  | |
| Housing Standard |  | |
| Infrastructure |  | |
| Health system |  | |
| Water resources |  | |
| Energy source |  | |
| Environmental state and pressure |  | |
| Vector reservoir |  | |
| Political structure |  | |
| Actors influencing disease event |  | |
| ***Exploring external shocks*** | | |
| Climate |  | |
| Conflict |  | |
| Economic |  | |
| ***Plausible Health Policies*** | | |
| Education on avoidance |  | |
| Nutrition |  | |
| Prevention and control intervention |  | |
| Control measures |  | |
| Treatments |  | |
| ***Vision*** | | |
| On site diagnostics and treatment | |  |
| Predictive models and information system(Surveillance feedback) | |  |
| Vaccination and other preventive actions | |  |
| Infrastructure change for resilience | |  |
| Economic measures for resilience | |  |
| Mitigate development risk drivers | |  |
| Agri-nutrition measures for health | |  |
| Security | |  |
| Environmental protection | |  |
